# Cancer screening inequities in a time of primary care reform: a population-based longitudinal study in Ontario, Canada

**DOI:** 10.1186/s12875-018-0827-1

**Published:** 2018-08-29

**Authors:** Aisha K. Lofters, Amy Mark, Monica Taljaard, Michael E. Green, Richard H. Glazier, Simone Dahrouge

**Affiliations:** 1 Department of Family & Community Medicine, St. Michael’s Hospital, 30 Bond St, Toronto, ON M5B1W8 Canada; 2 Department of Family & Community Medicine, University of Toronto, 500 University Ave. 5th Floor, Toronto, ON M5G1V7 Canada; 3 Centre for Urban Health Solutions, Li Ka Shing Knowledge Institute, St. Michael’s Hospital, 30 Bond St, Toronto, ON M5B1W8 Canada; 4 Dalla Lana School of Public Health, University of Toronto, 155 College St. 6th Floor, Toronto, ON M5T3M7 Canada; 5 Institute for Clinical Evaluative Sciences, G1 06, 2075 Bayview Ave, Toronto, ON M4N3M5 Canada; 6 Ottawa Hospital Research Institute, 501 Smyth Box 511, Ottawa, ON K1H 8L6 Canada; 7 Department of Family Medicine, Queen’s University, 220 Bagot St, Kingston, ON K7L5E9 Canada; 8 CT Lamont Primary Health Care Research Centre, Bruyere Research Institute, 43 Bruyère St, Ottawa, ON K1N5C8 Canada; 9 Department of Family Medicine, University of Ottawa, 600 Peter Morand Crescent Suite 201, Ottawa, ON K1G 5Z3 Canada

**Keywords:** Cancer screening, Primary care, Health equity, Primary care reform, Health disparities

## Abstract

**Background:**

Primary care has been reformed in recent years in Ontario, Canada, with a move away from traditional fee-for-service to enhanced fee-for-service and capitation-based models. It is unclear how new models have affected disparities in cancer screening. We evaluated whether Ontario’s enhanced fee-for-service model was associated with a change in the gaps in cancer screening for people living with low income and people who are foreign-born.

**Methods:**

We conducted a population-based longitudinal analysis from 2002 to 2013 of Ontario family physicians who transitioned from traditional fee-for-service to enhanced fee-for-service. The binary outcomes of interest were adherence to cervical, breast and colorectal cancer screening recommendations. Outcomes were analyzed using mixed-effects logistic regression. Analyses produced annual odds ratios comparing the odds of being up-to-date for screening among patients in enhanced fee-for-service versus patients in traditional fee-for-service for each social stratum separately. We calculated the ratios of stratum-specific odds ratios to assess whether the transition from traditional to enhanced fee-for-service was associated with a change in screening gaps between immigrants and long-term residents, and between people in the lowest and highest neighbourhood income quintiles.

**Results:**

Throughout the study period, cancer screening was consistently lower among immigrants and among people in the lowest income quintile. Transition to enhanced fee-for-service was generally associated with increased screening uptake for all, however for most years, ratios of ratios were significantly less than 1 for all three cancer screening types, indicating that there was a widening of the screening gap between immigrants and long-term residents and between people living in the lowest vs. highest income quintile associated with transitions.

**Conclusion:**

The transition to enhanced fee-for-service in Ontario was generally associated with a widening of screening inequities for foreign-born and low-income patients.

## Background

Screening for cancers, specifically cervical, breast and colorectal cancer (CRC), are integral components of primary care, with proven benefits for cancer morbidity and mortality. For example, for CRC screening, it has been estimated that if 70% of screen-eligible adults were screened regularly with either a biennial fecal occult blood test or a colonoscopy every ten years, CRC mortality could decrease by as much as 15% [1–5]. Mortality for cervical cancer has also dropped precipitously in Canada over recent decades to among the world’s lowest due to regular screening [1–3]. However, despite a universal healthcare system and organized provincial screening programs [6–9], screening inequalities for cervical, breast, and colorectal cancer (CRC) are well documented in Ontario — Canada’s most populous province. People living in low-income neighbourhoods and of low socioeconomic status have lower screening participation rates than their more advantaged peers for all three cancers [10–19]. Similarly, foreign-born persons are more likely to be under-screened for all three cancers, with certain immigrant groups being particularly vulnerable [10, 11, 14, 15, 20–22]. Barriers to screening are numerous, multi-level and complex, and exist at the patient, provider and healthcare system level [23–28].

Primary care reform in Ontario began in 2002, with the aims of expanding access to primary care, increasing emphasis on disease prevention, and improving quality of care [29–31]. Practices could transition from traditional fee-for-service to capitation-based payment models, wherein the majority of physician remuneration is based on the number and profile of patients under their care, or maintain their fee-for-service (FFS) payment structure but transition to an enhanced model of care, often referred to as enhanced fee-for-service (Table 1). Key elements of these new models were formalized patient enrolment (hence their name attribution “Patient Enrolment Model” or PEM) and financial incentives and bonuses, including for screening for cervical, breast and colorectal cancer for enrolled patients [29, 31, 32]. Adoption of the reform models was a success in that, by 2011, 77% of Ontario residents were enrolled with a physician working in a PEM [29, 32, 33]. However, it is unclear whether and how the new PEMs models affected existing gaps in care across social strata.

**Table 1:** Primary Care Models in Ontario

Primary Care Model Types	Core Features
Traditional fee-for-service^a^	No enrolment of patients. Fee-for-service payment for services rendered
Enhanced fee-for-service^a^	Enrolment of patients encouraged. Fee-for-service payment for services rendered. Incentives and bonuses (including. For achieving cancer screening targets) for enrolled patients. Escalating cancer screening bonuses for achieving set targets start if at least 60% of enrolled eligible women up-to-date on cervical screening, if at least 55% of enrolled eligible women up-to-date on breast screening, or if at least 15% of enrolled eligible adults up-to-date on colorectal screening.
Capitation-based	Enrolment of patients required. Capitation payment covering a defined basket of services, with 15% of the usual fee-for-service when these services are rendered and 100% fee-for-service for services outside the basket. Incentives and bonuses (including for achieving cancer screening targets as above) for enrolled patients
Capitation-based team model	As above, including funding to create interdisciplinary teams
Community health centres	Interdisciplinary teams, serve harder-to-reach populations, salaried payment

^a^Models of interest in this study

We undertook this study to evaluate whether transition to the enhanced FFS model had an impact on the known gaps in cancer screening by immigration status and across socioeconomic strata. We hypothesized that transition to the enhanced FFS model would be associated with a reduction in screening inequities. We limited our evaluation to practices having transitioned from the traditional FFS model to the enhanced FFS model as the other PEMs also include significant payment reform in addition to the incentives and bonuses.

## Methods

### Design

We conducted a population-based longitudinal analysis from 2002 to 2013 of family physicians who transitioned from the traditional FFS to the enhanced FFS model during that time period in Ontario.

### Study setting and context

Ontario is Canada’s most populous province, with 13.8 million current residents, 28.5% of whom were born outside Canada [34]. Ontario’s cervical and breast screening programs were established well before the study period: in 1997 and 1990 respectively [26], but ColonCancerCheck, Ontario’s colorectal cancer screening program, was launched with a public awareness campaign in 2008 [35]. Cervical cancer screening guidelines changed in 2012, from a recommendation of annual Pap tests followed by screening every two to three years after three consecutive normal results, to a recommendation of screening every three years [36]. Current provincial cancer screening recommendations are at least one Pap test in the past three years for cervical cancer, at least one mammogram in the past two years for breast cancer, and at least one fecal occult blood test in the past two years for CRC screening. People are also considered up-to-date on CRC screening if they have had at least one colonoscopy in the past ten years or at least one sigmoidoscopy or barium enema in the past five years [6–9].

### Data sources

The following datasets were linked using unique encoded identifiers and analyzed at the Institute for Clinical Evaluative Sciences (ICES). The Registered Persons Database (RPDB) includes the age, sex and postal code of all Ontario residents who are eligible for the universal Ontario Health Insurance Plan (OHIP). The OHIP database contains procedural and diagnostic codes claimed by physicians in the province. The Immigration, Refugees and Citizenship Canada (IRCC) database consists of demographic information on Ontario’s immigrants and refugees recorded on the date of issue of the landing visa, going back to 1985. The Ontario Cancer Registry documents all Ontario residents who have been newly diagnosed with, or died of, cancer. The Canadian Institute for Health Information Discharge Abstract Database contains clinical data from hospital discharges. The Ontario Breast Screening Program database contains data on screening mammograms. The Client Agency Program Enrolment (CAPE) database is updated bimonthly and identifies all Ontarians who are enrolled with a physician in a PEM, and the Corporate Provider Database records which family physicians participate in these PEMs. The Institute for Clinical Evaluative Sciences’ Physicians’ Database records demographic information about Ontario’s physicians who are in active practice. This study was approved by the institutional review board at Sunnybrook Health Sciences Centre, Toronto, Canada.

### Study population – Physicians and patients

We created an open cohort that included all family physicians who provided comprehensive primary care in Ontario and who transitioned from traditional FFS to enhanced FFS at some point between 2002 and 2011, and we followed physicians until 2013. Only physicians for whom we had at least two years pre and post transition data were retained in the analysis i.e. physicians who transitioned to another model type within two years were excluded.

We followed physicians longitudinally and established their corresponding patient rosters annually. Patients were attributed to individual physicians by using the CAPE database. Patients not formally enrolled with a primary care physician (including all patients prior to transition) were attributed by virtual enrolment i.e. they were attributed to the physician providing the most care financially to that patient in the two years prior to the end of each fiscal year based on a set of core primary care fee codes [16, 37–40]. In any given year, the patient dataset was further limited to individuals who were eligible for cervical, breast or CRC screening at the start of the two-year period preceding that year. We followed the provincial recommendations for screening and prevention to determine appropriateness of care [6–9]. To be eligible for cervical screening, women had to be 21 to 69 years of age and have no prior hysterectomy or prior diagnosis of invasive cervical cancer, endometrial cancer or ovarian cancer. We chose to apply the post-2012 cervical guidelines throughout the entire study period, as a three-year screening interval was also acceptable in the older guidelines. Women eligible for breast cancer screening were 50 to 69 years of age and had no prior diagnosis of invasive breast cancer. Patients considered eligible for CRC screening were 50 to 74 years of age and had no prior diagnoses of invasive CRC or inflammatory bowel disease.

### Outcomes

The binary outcomes of interest were adherence to screening recommendations. Outcomes were measured annually at the patient level and reflected whether a patient was up-to-date on cervical, breast, or CRC screening. This was done separately for each type of screening that a patient was eligible for. At any point in time, the look-back window for outcome ascertainment was the two or three preceding years, depending on the recommendations. For example, a cervical screening manoeuvre conducted in any particular year would lead to a classification as up to date for that year and the next two years.

### Subgroup definitions

The main subgroups of interest for the analysis were immigrant status and socio-economic strata. We identified immigrant patients as all those who were included in the IRCC database. Long-term residents of the province were defined as those not included in the IRCC database. These consisted of Canadian-born residents but also included immigrants who had landed prior to 1985. To define socioeconomic strata, we used the Postal Code Conversion File and Statistics Canada 2006 Census data to determine the neighbourhood income quintiles based on patients’ yearly postal code in the RPDB. For the purpose of the statistical analysis, we focused on the lowest versus highest income quintiles.

### Statistical analysis

The unit of analysis was the individual patient, but inferences were made at the provider level. Outcomes were analyzed using mixed-effects logistic regression, estimated using the method of pseudo-likelihood based on linearization. The practice was modelled as a random effect to account for correlation among multiple patients belonging to the same practice. The main independent variable was a time-varying indicator, defined as the type of practice (traditional or enhanced FFS). As preliminary analyses demonstrated the presence of a strong secular trend (changes over time in screening rates prior to any transition) as well as differences in the observed effects of the transition by year, our model included fixed categorical terms for fiscal year and the interaction between fiscal year and the type of practice. Thus, our statistical models allowed comparisons between physicians who transitioned to enhanced FFS and physicians who remained behind in traditional FFS in each study year where transitions occurred.

To examine our primary questions of whether gaps in cancer screening across immigration status changed with the transition to enhanced FFS, we included two-way interaction terms between practice type and year, as well as three-way interaction terms between practice type, year and immigrant status. This analysis was then repeated for income quintile. The analyses produced annual odds ratios comparing the odds of being up to date for screening among patients in enhanced FFS versus patients in traditional FFS for each social stratum separately. We then calculated the ratios of these stratum-specific odds ratios, to directly assess whether the transition from traditional to enhanced FFS was associated with a narrowing or widening of the screening gaps between immigrants and long-term residents, and between people in the lowest and highest income quintiles. Traditional FFS practices, long-term residents, and people living in the highest income quintile were used as the reference groups for ratios of ratios. Accordingly, a ratio of odds ratios less than 1 indicated that the transition from traditional FFS to enhanced FFS was associated with a widening of screening gaps, while a ratio of odds ratios greater than 1 indicated that the transition was associated with a narrowing of screening gaps. We used the 95% confidence intervals to determine whether these ratios were statistically significant, whereby a confidence interval exclusive of 1 indicated a significant narrowing or widening of the screening gap.

To account for potential confounders (provider and patient characteristics associated with both the type of FFS model and screening outcomes), all our analyses adjusted for the following physician characteristics: sex, international medical graduate status, years since graduation, panel size and rurality, and patient characteristics: age, sex, co-morbidities and rurality. Rurality was determined based on the Rurality Index of Ontario score [41], and the level of co-morbidity was categorized using the Johns Hopkins Adjusted Clinical Groups Case-Mix System [42].

All statistical tests were performed at the two-sided 5% level of significance, using SAS for Unix, version 9.1.3 (SAS Institute, Cary, NC).

## Results

During the study period, 7336 family physicians transitioned from the traditional FFS primary care model to a PEM. Most of these (6145) adopted the enhanced FFS model initially, with 5268 (86%) doing so from 2004 to 2007. In our cohort, we excluded physicians who transitioned back to traditional FFS [25], with less than two years of data post-transition (375), and who did not offer comprehensive primary care (412). There were 4670 physicians in our cohort in 2002 (Table 2). By 2013, there were 2811 physicians remaining in the cohort i.e. 2811 physicians who had not gone on to transition to another PEM type. The number of patients enrolled to these physicians and who were eligible for each type of cancer screening decreased from 2002 to 2013, in line with the decrease in the absolute number of physicians. However, the proportion of patients who were foreign-born increased.

**Table 2:** Relative distributions of subgroups included in analyses in 2002 (first year of study period) and 2013 (last year of study period) by the screening type for which they were eligible

		2002	2013
Number of physicians included in analysis		4 670	2 181
Cervical cancer screening	Number of patients eligible for screening	2 004 009	1 127 283
	Number (%) of screen-eligible patients classified as immigrants	297 501 (14.9)	356 562 (31.6)
	Number (%) of screen-eligible patients in income quintile		
	Quintile 1 (lowest)	358 094 (17.9)	212 638 (18.9)
	Q2	390 558 (19.5)	229 899 (20.4)
	Q3	411 025 (20.5)	236 375 (21.0)
	Q4	423 745 (21.1)	240 414 (21.3)
	Q5 (highest)	420 587 (21.0)	207 957 (18.5)
Breast cancer screening	Number of patients eligible for screening	560 047	407 625
	Number (%) of screen-eligible patients classified as immigrants	49 583 (8.9%)	91 766 (22.5%)
	Number (%) of screen-eligible patients in income quintile		
	Q1 (lowest)	94 447 (16.9%)	71 173 (17.5%)
	Q2	108 869 (19.4%)	82 187 (20.2%)
	Q3	114 438 (20.4%)	84 578 (20.8%)
	Q4	116 199 (20.8%)	87 072 (21.4%)
	Q5 (highest)	126 094 (22.5%)	82 615 (20.3%)
Colorectal cancer screening	Number of patients eligible for screening	1 344 891	925 961
	Number (%) of screen-eligible patients classified as immigrants	108 852 (8.1%)	206 705 (22.3%)
	Number (%) of screen-eligible patients in income quintile		
	Q1 (lowest)	221 906 (16.5%)	160 155 (17.3%)
	Q2	259 256 (19.3%)	185 972 (20.1%)
	Q3	275 213 (20.5%)	192 221 (20.8%)
	Q4	281 479 (20.9%)	198 951 (21.5%)
	Q5 (highest)	307 037 (22.8%)	188 662 (20.4%)

**Fig. 1:**
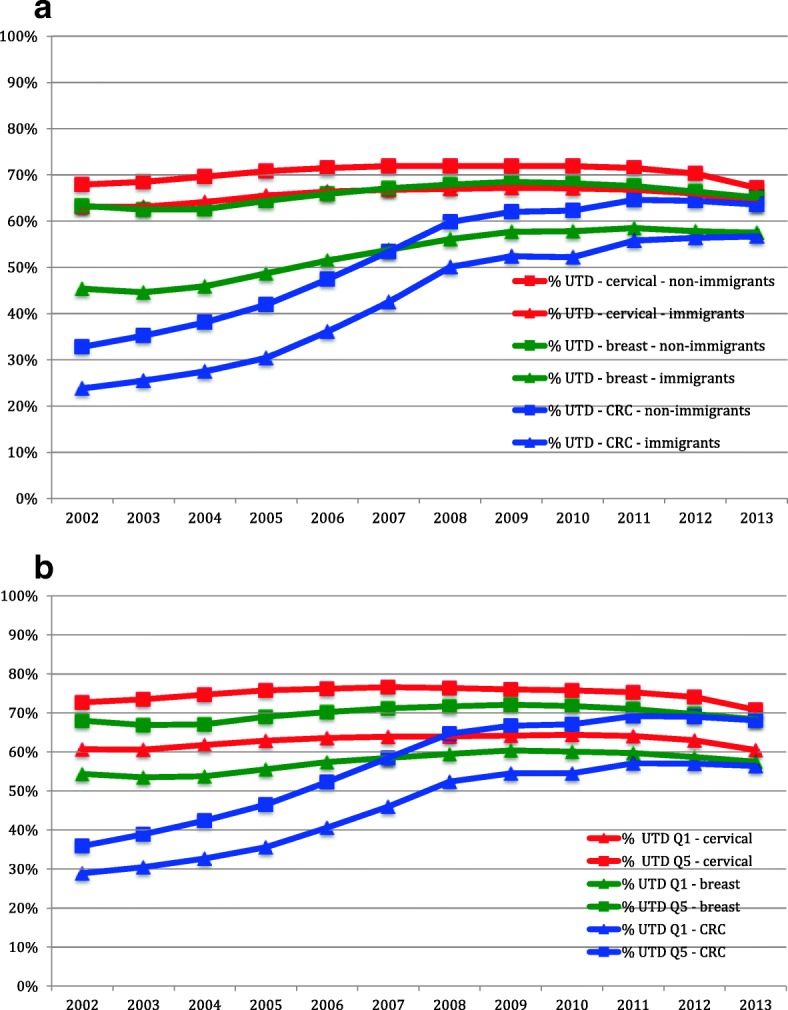
Screening across time by fiscal year. UTD = proportion of patients up-to-date on that screening type for that fiscal year as defined by provincial guidelines. CRC = colorectal cancer. **a** Stratified by immigrant status. **b** Stratified by income quintiles

By the end of the study period, screening rates for all included patients were 66.4% (cervical screening), 64.0% (breast screening) and 62.6% (CRC screening). Throughout that period, screening was consistently lower among immigrants and among people in the lowest income quintile when compared to long-term residents and people in the highest income quintile respectively (Figs. 1a-b). CRC screening rates increased sharply from 2002. The gap in breast cancer screening between immigrants and long-term residents appeared to be reduced over time.

**Fig. 2:**
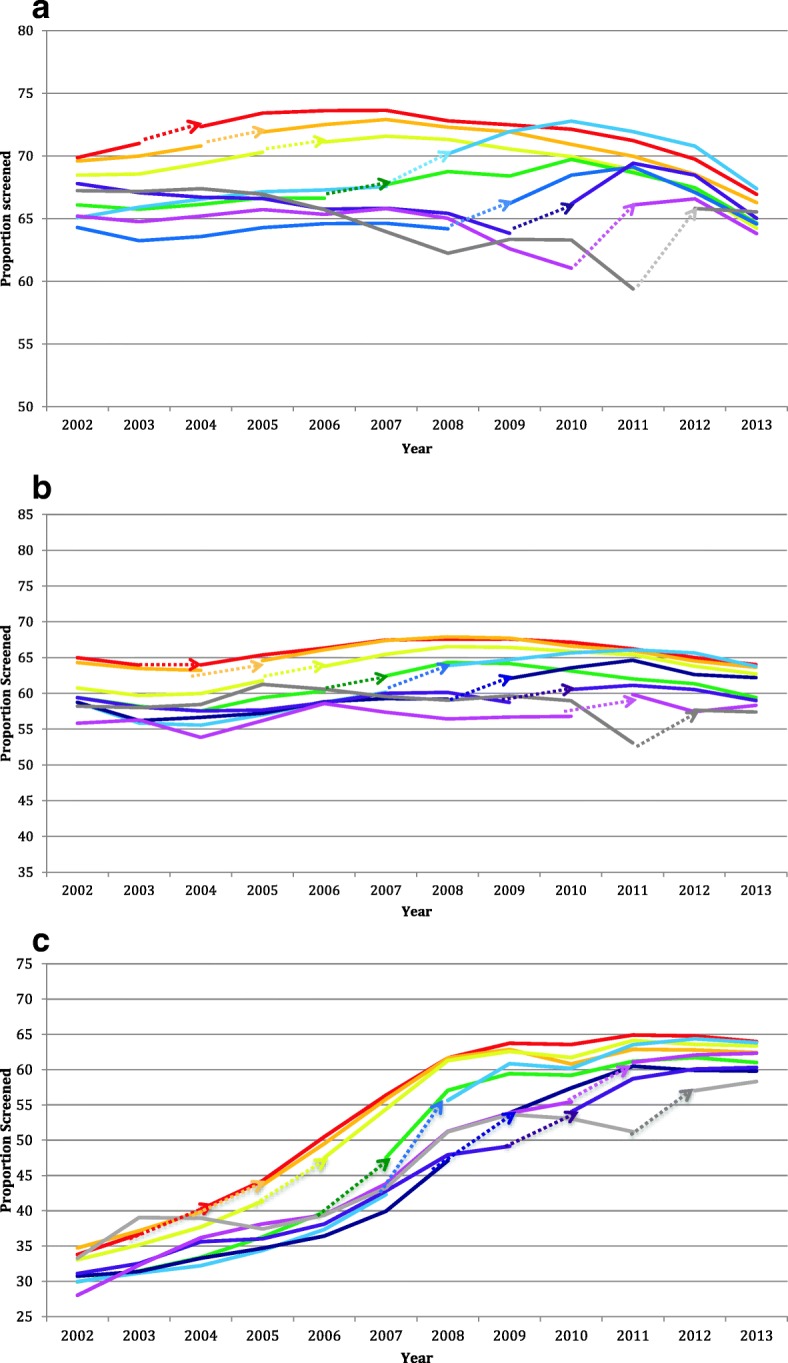
Proportion of patients up-to-date stratified by year of transition from traditional fee-for-service to enhanced fee-for-service. Dotted arrows indicate the fiscal year of transition for practices. **a** Cervical cancer Screening. **b** Breast cancer screening. **c** Colorectal cancer screening

Figures 2a-c depict the observed rates of cervical, breast and CRC screening by fiscal year, stratified by the year of transition. Family physicians who were early adopters of the enhanced FFS model tended to have higher screening rates prior to transition, and the transition did not appear to introduce a change in either the level or the trend over time. In contrast, the late adopters tended to have lower screening rates at the time of transition and a greater increase in screening rates after transition. Overall, there was a drop at the end of the study period in cervical cancer screening uptake.

Tables 3, 4, 5 show unadjusted and adjusted odds ratios produced from the mixed-effects logistic regression analyses, comparing the enhanced to traditional FFS models. Odds ratios are presented for each year, separately for patients who were foreign-born and who were long-term residents. One covariate, physician panel size, was excluded from the breast cancer screening model as the model failed to converge with this covariate included. This variable had a very small effect in the other two fully adjusted models. For cervical and breast cancer screening, odds ratios for immigrants were less than 1 in earlier years (suggesting worse screening uptake in enhanced versus traditional FFS) and generally showed a steady increase to being greater than 1 in later years (suggesting better screening uptake in enhanced versus traditional FFS). Odds ratios were greater than 1 for patients who were long-term residents of the province throughout the study period suggesting better screening uptake in enhanced versus traditional FFS among long-term residents than among recent immigrants. For CRC screening, odds ratios for both immigrants and long-term residents were generally greater than 1 throughout the study period, suggesting better screening uptake in enhanced versus traditional FFS.

**Table 3:** Unadjusted and adjusted odds ratios comparing enhanced fee-for-service to traditional fee-for-service produced from the cervical cancer screening model

	Year	Unadjusted			Adjusted for Patient Characteristics			Adjusted for Patient and Physician Characteristics		
		Odds Ratio	95% CI	*p*-value	Odds Ratio	95% CI	*p*-value	Odds Ratio	95% CI	*p*-value
Immigrants										
Enhanced vs. traditional fee-for-service	2005	0.92	(0.90–0.93)	<.0001	0.89	(0.87–0.91)	<.0001	0.94	(0.92–0.96)	<.0001
	2006	0.97	(0.96–0.98)	<.0001	0.95	(0.94–0.97)	<.0001	1.00	(0.98–1.01)	0.567
	2007	0.93	(0.98–1.01)	0.404	0.98	(0.97–1.00)	0.059	1.04	(1.03–1.06)	<.0001
	2008	0.94	(0.92–0.96)	<.0001	0.95	(0.93–0.97)	<.0001	1.04	(1.02–1.06)	0.001
	2009	1.04	(1.01–1.06)	0.004	1.08	(1.06–1.11)	<.0001	1.14	(1.11–1.17)	<.0001
	2010	1.01	(0.98–1.04)	0.503	1.07	(1.04–1.10)	<.0001	1.15	(1.12–1.19)	<.0001
	2011	1.11	(1.07–1.16)	<.0001	1.16	(1.12–1.21)	<.0001	1.26	(1.21–1.31)	<.0001
	2012	1.12	(1.08–1.17)	<.0001	1.26	(1.20–1.31)	<.0001	1.25	(1.19–1.30)	<.0001
Long-Term Residents										
Enhanced vs. traditional fee-for-service	2005	1.04	(1.03–1.05)	<.0001	1.05	(1.04–1.06)	<.0001	1.05	(1.04–1.06)	<.0001
	2006	1.02	(1.02–1.03)	<.0001	1.04	(1.03–1.05)	<.0001	1.06	(1.05–1.07)	<.0001
	2007	1.08	(1.07–1.10)	<.0001	1.10	(1.08–1.11)	<.0001	1.13	(1.11–1.14)	<.0001
	2008	1.09	(1.07–1.10)	<.0001	1.13	(1.11–1.14)	<.0001	1.17	(1.15–1.19)	<.0001
	2009	1.18	(1.16–1.20)	<.0001	1.22	(1.19–1.24)	<.0001	1.20	(1.18–1.23)	<.0001
	2010	1.18	(1.16–1.21)	<.0001	1.25	(1.22–1.28)	<.0001	1.28	(1.24–1.31)	<.0001
	2011	1.12	(1.09–1.16)	<.0001	1.19	(1.15–1.23)	<.0001	1.24	(1.19–1.28)	<.0001
	2012	1.11	(1.07–1.15)	<.0001	1.23	(1.18–1.27)	<.0001	1.20	(1.16–1.25)	<.0001
Ratios of Odds Ratios										
Difference in enhanced vs. traditional fee-for-service between immigrants vs. long-term residents	2005	0.88	(0.86–0.90)	<.0001	0.85	(0.83–0.87)	<.0001	0.90	(0.88–0.92)	<.0001
	2006	0.95	(0.93–0.96)	<.0001	0.92	(0.90–0.93)	<.0001	0.94	(0.92–0.96)	<.0001
	2007	0.92	(0.90–0.94)	<.0001	0.90	(0.88–0.92)	<.0001	0.93	(0.91–0.95)	<.0001
	2008	0.86	(0.84–0.88)	<.0001	0.84	(0.82–0.86)	<.0001	0.89	(0.86–0.91)	<.0001
	2009	0.88	(0.85–0.91)	<.0001	0.89	(0.86–0.92)	<.0001	0.94	(0.92–0.97)	0.000
	2010	0.85	(0.22–0.89)	<.0001	0.86	(0.82–0.89)	<.0001	0.90	(0.87–0.94)	<.0001
	2011	0.99	(0.95–1.04)	0.794	0.98	(0.94–1.03)	0.433	1.02	(0.97–1.07)	0.477
	2012	1.01	(0.96–1.07)	0.607	1.02	(0.97–1.08)	0.393	1.04	(0.98–1.10)	0.180

Ratios of odds ratios are also presented, with long-term residents and traditional fee-for-service serving as referent groups

**Table 4:** Unadjusted and adjusted odds ratios comparing enhanced fee-for-service to traditional fee-for-service produced from the breast cancer screening model

	Year	Unadjusted			Adjusted for Patient Characteristics			Adjusted for Patient and Physician Characteristics		
		Odds Ratio	95% CI	*p*-value	Odds Ratio	95% CI	*p*-value	Odds Ratio	95% CI	*p*-value
Immigrants										
Enhanced vs. traditional fee-for-service	2005	0.86	(0.83–0.90)	<.0001	0.86	(0.82–0.90)	<.0001	0.89	(0.85–0.93)	<.0001
	2006	0.95	(0.92–0.98)	0.0016	0.96	(0.93–0.99)	0.0087	0.98	(0.94–1.01)	0.1181
	2007	0.98	(0.95–1.02)	0.3024	0.98	(0.95–1.02)	0.3391	1.02	(0.98–1.05)	0.4424
	2008	0.96	(0.92–1.00)	0.0656	0.97	(0.92–1.01)	0.1516	1.01	(0.97–1.06)	0.5703
	2009	0.99	(0.94–1.05)	0.7257	1.02	(0.97–1.08)	0.458	1.04	(0.98–1.10)	0.238
	2010	0.92	(0.86–0.98)	0.0082	0.95	(0.89–1.01)	0.0933	1.00	(0.93–1.06)	0.9135
	2011	1.09	(1.01–1.18)	0.0229	1.14	(1.06–1.23)	0.0008	1.21	(1.12–1.31)	<.0001
	2012	1.21	(1.11–1.32)	<.0001	1.30	(1.19–1.42)	<.0001	1.29	(1.18–1.40)	<.0001
Long-Term Residents										
Enhanced vs. traditional fee-for-service	2005	1.01	(1.00–1.02)	0.1411	1.01	(1.00–1.03)	0.0435	1.02	(1.00–1.03)	0.032
	2006	1.04	(1.02–1.05)	<.0001	1.04	(1.03–1.05)	<.0001	1.05	(1.04–1.07)	<.0001
	2007	1.09	(1.07–1.11)	<.0001	1.09	(1.07–1.11)	<.0001	1.11	(1.09–1.13)	<.0001
	2008	1.13	(1.10–1.16)	<.0001	1.14	(1.11–1.17)	<.0001	1.17	(1.14–1.20)	<.0001
	2009	1.19	(1.15–1.22)	<.0001	1.20	(1.17–1.24)	<.0001	1.19	(1.15–1.23)	<.0001
	2010	1.16	(1.11–1.21)	<.0001	1.17	(1.13–1.22)	<.0001	1.19	(1.14–1.24)	<.0001
	2011	1.20	(1.13–1.26)	<.0001	1.21	(1.15–1.27)	<.0001	1.25	(1.18–1.31)	<.0001
	2012	1.14	(1.07–1.21)	<.0001	1.19	(1.12–1.26)	<.0001	1.17	(1.10–1.24)	<.0001
Ratios of Odds Ratios										
Difference in enhanced vs. traditional fee-for-service between immigrants vs. long-term residents	2005	0.85	(0.82–0.89)	<.0001	0.85	(0.81–0.89)	<.0001	0.87	(0.84–0.91)	<.0001
	2006	0.92	(0.89–0.95)	<.0001	0.92	(0.89–0.95)	<.0001	0.93	(0.90–0.96)	<.0001
	2007	0.90	(0.86–0.94)	<.0001	0.90	(0.87–0.94)	<.0001	0.92	(0.88–0.96)	<.0001
	2008	0.85	(0.81–0.89)	<.0001	0.85	(0.80–0.89)	<.0001	0.87	(0.82–0.91)	<.0001
	2009	0.83	(0.78–0.89)	<.0001	0.85	(0.80–0.90)	<.0001	0.87	(0.82–0.93)	<.0001
	2010	0.79	(0.73–0.85)	<.0001	0.81	(0.75–0.87)	<.0001	0.84	(0.78–0.90)	<.0001
	2011	0.91	(0.84–1.00)	0.050	0.95	(0.86–1.03)	0.218	0.97	(0.89–1.07)	0.5267
	2012	1.06	(0.96–1.18)	0.273	1.10	(0.99–1.22)	0.089	1.10	(0.99–1.22)	0.0803

Ratios of odds ratios are also presented, with long-term residents and traditional fee-for-service serving as referent groups

**Table 5:** Unadjusted and adjusted odds ratios comparing enhanced fee-for-service to traditional fee-for-service produced from the colorectal cancer screening model

	Year	Unadjusted			Adjusted for Patient Characteristics			Adjusted for Patient and Physician Characteristics		
		Odds Ratio	95% CI	*p*-value	Odds Ratio	95% CI	*p*-value	Odds Ratio	95% CI	*p*-value
Immigrants										
Enhanced vs. traditional fee-for-service	2005	0.91	(0.89–0.94)	<.0001	0.91	(0.88–0.94)	<.0001	0.94	(0.91–0.97)	<.0001
	2006	1.02	(0.99–1.04)	0.1809	1.03	(1.01–1.05)	0.0074	1.04	(1.01–1.06)	0.0021
	2007	1.21	(1.18–1.24)	<.0001	1.22	(1.19–1.26)	<.0001	1.24	(1.21–1.28)	<.0001
	2008	1.13	(1.09–1.16)	<.0001	1.15	(1.11–1.19)	<.0001	1.19	(1.15–1.23)	<.0001
	2009	1.19	(1.14–1.23)	<.0001	1.23	(1.19–1.28)	<.0001	1.23	(1.19–1.28)	<.0001
	2010	0.99	(0.95–1.04)	0.7596	1.03	(0.98–1.07)	0.2438	1.06	(1.02–1.11)	0.007
	2011	1.06	(1.01–1.11)	0.0319	1.09	(1.04–1.15)	0.0012	1.13	(1.07–1.19)	<.0001
	2012	1.25	(1.18–1.32)	<.0001	1.38	(1.31–1.47)	<.0001	1.38	(1.30–1.57)	<.0001
Long-Term Residents										
Enhanced vs. traditional fee-for-service	2005	1.00	(0.99–1.01)	0.5301	1.00	(0.99–1.01)	0.528	1.00	(1.00–1.01)	0.4123
	2006	1.09	(1.08–1.10)	<.0001	1.10	(1.09–1.10)	<.0001	1.10	(1.09–1.11)	<.0001
	2007	1.25	(1.23–1.26)	<.0001	1.26	(1.24–1.27)	<.0001	1.27	(1.25–1.28)	<.0001
	2008	1.20	(1.18–1.22)	<.0001	1.23	(1.21–1.25)	<.0001	1.25	(1.23–1.27)	<.0001
	2009	1.27	(1.25–1.30)	<.0001	1.31	(1.28–1.34)	<.0001	1.29	(1.26–1.32)	<.0001
	2010	1.15	(1.12–1.18)	<.0001	1.17	(1.14–1.20)	<.0001	1.19	(1.16–1.22)	<.0001
	2011	1.10	(1.06–1.14)	<.0001	1.11	(1.07–1.15)	<.0001	1.14	(1.10–1.18)	<.0001
	2012	1.09	(1.05–1.14)	<.0001	1.17	(1.12–1.21)	<.0001	1.15	(1.11–1.20)	<.0001
Ratios of Odds Ratios										
Difference in enhanced vs. traditional fee-for-service between immigrants vs. long-term residents	2005	0.92	(0.89–0.94)	<.0001	0.91	(0.88–0.94)	<.0001	0.94	(0.91–0.96)	<.0001
	2006	0.93	(0.91–0.95)	<.0001	0.94	(0.92–0.963)	<.0001	0.94	(0.92–0.96)	<.0001
	2007	0.97	(0.95–1.00)	0.0508	0.97	(0.95–1.00)	0.0659	0.98	(0.95–1.01)	0.1798
	2008	0.94	(0.90–0.97)	0.0002	0.93	(0.90–0.97)	<.0001	0.95	(0.91–0.98)	0.0031
	2009	0.93	(0.89–0.97)	0.0009	0.94	(0.90–0.98)	0.0061	0.96	(0.92–1.00)	0.037
	2010	0.87	(0.82–0.91)	<.0001	0.88	(0.84–0.93)	<.0001	0.89	(0.85–0.94)	<.0001
	2011	0.96	(0.90–1.02)	0.1821	0.99	(0.93–1.05)	0.671	0.99	(0.93–1.06)	0.8325
	2012	1.14	(1.07–1.22)	0.0001	1.19	(1.11–1.27)	<.0001	1.20	(1.12–1.29)	<.0001

Ratios of odds ratios are also presented, with long-term residents and traditional fee-for-service serving as referent groups

Tables 3, 4, 5 also display ratios of odds ratios. For most years (2005–2010), ratios of ratios were significantly less than 1 for all three cancer screening types, indicating that there was a widening of the screening gap between immigrants and long-term residents associated with transitions in those years. From 2011 onward, the ratios of ratios were no longer statistically significant, except for CRC screening where the ratios of both unadjusted and adjusted odds ratios changed direction and were significantly greater than 1 in 2012, indicating that by the end of the observation period, transition was associated with a significantly smaller screening gap for immigrants (ratio of odds ratios 1.199 [95% CI 1.118–1.287] adjusted for patient and physician characteristics).

In 2002, when all physicians were still in traditional FFS, fully adjusted odds ratios comparing immigrant patients to long-term residents were 0.876 [95% CI 0.868–0.884] for cervical screening, 0.639 [95% confidence interval 0.626–0.653] for breast screening, and 0.728 [95% CI 0.716–0.740] for CRC screening. In 2013, at which point all physicians had transitioned to enhanced FFS, fully adjusted odds ratios comparing immigrant to long-term residents had risen to 0.908 [95% CI 0.900–0.916] for cervical screening, 0.913 [95% CI 0.898–0.927] for breast screening, and 0.881 [95% confidence interval 0.872–0.890] for CRC screening (data not shown).

Tables 6, 7, 8 show both unadjusted and adjusted odds ratios produced from the regression analyses, comparing patients living in the lowest and highest income quintiles and stratified by year and model type (data for income quintiles Q2-Q4 not shown). For all three types of cancer screening, odds ratios comparing enhanced to traditional FFS were generally greater than 1 for patients in both Q1 and Q5, indicating better screening uptake in enhanced versus traditional models.

**Table 6:** Unadjusted and adjusted odds ratios comparing enhanced to traditional fee-for-service produced from the cervical cancer screening model

	Year	Unadjusted			Adjusted for Patient Characteristics			Adjusted for Patient and Physician Characteristics		
		Odds Ratio	95% CI	*p*-value	Odds Ratio	95% CI	*p*-value	Odds Ratio	95% CI	*p*-value
Income Q1 (lowest)										
Enhanced vs. traditional fee-for-service	2005	0.97	(0.96–0.99)	0.000	0.96	(0.95–0.98)	<.0001	0.98	(0.97–1.00)	0.0231
	2006	0.99	(0.98–1.00)	0.080	0.98	(0.96–0.99)	0.0008	1.00	(0.99–1.02)	0.7417
	2007	1.03	(1.01–1.05)	0.004	1.02	(1.00–1.04)	0.0271	1.06	(1.04–1.08)	<.0001
	2008	0.98	(0.96–1.01)	0.145	0.99	(0.97–1.0)	0.649	1.04	(1.02–1.07)	0.001
	2009	1.10	(1.07–1.14)	<.0001	1.14	(1.10–1.17)	<.0001	1.16	(1.13–1.20)	<.0001
	2010	1.04	(1.00–1.08)	0.028	1.10	(1.06–1.14)	<.0001	1.16	(1.11–1.20)	<.0001
	2011	1.04	(1.00–1.09)	0.082	1.09	(1.04–1.15)	0.0004	1.19	(1.31–1.25)	<.0001
	2012	1.08	(1.02–1.14)	0.006	1.21	(1.14–1.28)	<.0001	1.20	(1.13–1.27)	<.0001
Income Q5 (highest)										
Enhanced vs. traditional fee-for-service	2005	1.06	(1.04–1.07)	<.0001	1.07	(1.05–1.09)	<.0001	1.06	(1.05–1.08)	<.0001
	2006	1.04	(1.03–1.06)	<.0001	1.06	(1.05–1.08)	<.0001	1.09	(1.08–1.11)	<.0001
	2007	1.15	(1.13–1.18)	<.0001	1.18	(1.15–1.21)	<.0001	1.20	(1.17–1.23)	<.0001
	2008	1.15	(1.12–1.19)	<.0001	1.20	(1.17–1.24)	<.0001	1.26	(1.22–1.30)	<.0001
	2009	1.18	(1.14–1.22)	<.0001	1.24	(1.20–1.29)	<.0001	1.24	(1.20–1.29)	<.0001
	2010	1.26	(1.20–1.32)	<.0001	1.35	(1.29–1.42)	<.0001	1.38	(1.31–1.44)	<.0001
	2011	1.21	(1.15–1.29)	<.0001	1.33	(1.25–1.41)	<.0001	1.37	(1.29–1.45)	<.0001
	2012	1.17	(1.10–1.25)	<.0001	1.33	(1.24–1.42)	<.0001	1.31	(1.23–1.41)	<.0001
Ratios of Odds Ratios										
Difference in enhanced vs. traditional fee-for-service between Q1 vs. Q5	2005	0.92	(0.90–0.94)	<.0001	0.90	(0.88–0.92)	<.0001	0.92	(0.90–0.95)	<.0001
	2006	0.95	(0.93–0.97)	<.0001	0.92	(0.90–0.94)	<.0001	0.92	(0.90–0.94)	<.0001
	2007	0.89	(0.87–0.92)	<.0001	0.87	(0.84–0.89)	<.0001	0.88	(0.86–0.909)	<.0001
	2008	0.85	(0.82–0.89)	<.0001	0.83	(0.79–0.86)	<.0001	0.83	(0.80–0.86)	<.0001
	2009	0.94	(0.90–0.98)	0.007	0.92	(0.87–0.96)	0.000	0.94	(0.89–0.98)	0.0059
	2010	0.83	(0.78–0.88)	<.0001	0.81	(0.76–0.86)	<.0001	0.84	(0.79–0.89)	<.0001
	2011	0.86	(0.80–0.92)	<.0001	0.82	(0.76–0.89)	<.0001	0.87	(0.81–0.94)	0.0003
	2012	0.92	(0.85–1.01)	0.064	0.91	(0.84–0.99)	0.032	0.91	(0.83–0.99)	0.0353

Results for Q2-Q4 not shown. Ratios of odds ratios are also presented, with Q5 and traditional fee-for-service serving as referent groups

**Table 7:** Unadjusted and adjusted odds ratios comparing enhanced to traditional fee-for-service produced from the breast cancer screening model

	Year	Unadjusted			Adjusted for Patient Characteristics			Adjusted for Patient and Physician Characteristics		
		Odds Ratio	95% CI	*p*-value	Odds Ratio	95% CI	*p*-value	Odds Ratio	95% CI	*p*-value
Income Q1 (lowest)										
Enhanced vs. traditional fee-for-service	2005	0.97	(0.95–1.00)	0.0438	0.97	(0.94–0.99)	0.0123	0.97	(0.95–1.00)	0.0646
	2006	1.02	(1.00–1.04)	0.1186	1.01	(0.98–1.03)	0.5442	1.02	(0.99–1.04)	0.2084
	2007	1.05	(1.01–1.08)	0.0141	1.03	(0.99–1.06)	0.1498	1.04	(1.01–1.08)	0.0165
	2008	1.06	(1.01–1.11)	0.0169	1.06	(1.01–1.11)	0.0187	1.08	(1.03–1.14)	0.0009
	2009	1.07	(1.01–1.14)	0.0147	1.10	(1.04–1.16)	0.0017	1.10	(1.04–1.16)	0.0015
	2010	0.99	(0.92–1.06)	0.8048	1.01	(0.94–1.09)	0.7935	1.04	(0.97–1.12)	0.2686
	2011	1.07	(0.98–1.17)	0.1351	1.09	(0.99–1.19)	0.0711	1.14	(1.04–1.25)	0.0043
	2012	1.11	(1.00–1.23)	0.0551	1.16	(1.04–1.29)	0.0058	1.15	(1.03–1.28)	0.0117
Income Q5 (highest)										
Enhanced vs. traditional fee-for-service	2005	1.03	(1.00–1.05)	0.027	1.04	(1.01–1.06)	0.0033	1.03	(1.01–1.06)	0.0135
	2006	1.03	(1.01–1.05)	0.0167	1.04	(1.02–1.07)	0.0002	1.06	(1.03–1.08)	<.0001
	2007	1.11	(1.07–1.15)	<.0001	1.11	(1.07–1.16)	<.0001	1.13	(1.09–1.17)	<.0001
	2008	1.14	(1.09–1.2)	<.0001	1.15	(1.09–1.21)	<.0001	1.18	(1.12–1.24)	<.0001
	2009	1.20	(1.13–1.28)	<.0001	1.22	(1.15–1.30)	<.0001	1.21	(1.14–1.29)	<.0001
	2010	1.22	(1.13–1.32)	<.0001	1.24	(1.14–1.34)	<.0001	1.25	(1.15–1.36)	<.0001
	2011	1.26	(1.14–1.40)	<.0001	1.29	(1.17–1.43)	<.0001	1.33	(1.20–1.47)	<.0001
	2012	1.14	(1.02–1.28)	0.0276	1.19	(1.06–1.35)	0.0035	1.19	(1.05–1.34)	0.005
Ratios of Odds Ratios										
Difference in enhanced vs. traditional fee-for-service between Q1 vs. Q5	2005	0.95	(0.91–0.98)	0.002	0.93	(0.90–0.97)	<.0001	0.94	(0.91–0.98)	0.002
	2006	0.99	(0.96–1.02)	0.594	0.97	(0.94–1.00)	0.032	0.96	(0.93–0.99)	0.014
	2007	0.94	(0.90–0.99)	0.018	0.92	(0.88–0.97)	0.002	0.93	(0.88–0.97)	0.003
	2008	0.93	(0.87–0.99)	0.029	0.92	(0.86–0.99)	0.018	0.92	(0.86–0.98)	0.016
	2009	0.89	(0.82–0.97)	0.006	0.90	(0.83–0.98)	0.011	0.91	(0.84–0.99)	0.020
	2010	0.81	(0.73–0.90)	0.000	0.82	(0.73–0.91)	0.000	0.83	(0.75–0.93)	0.001
	2011	0.85	(0.74–0.97)	0.014	0.84	(0.74–0.96)	0.011	0.86	(0.75–0.98)	0.027
	2012	0.97	(0.83–1.14)	0.716	0.97	(0.83–1.14)	0.734	0.97	(0.82–1.13)	0.671

Results for Q2-Q4 not shown. Ratios of odds ratios are also presented, with Q5 and traditional fee-for-service serving as referent groups

**Table 8:** Unadjusted and adjusted odds ratios comparing enhanced to traditional fee-for-service produced from the CRC screening model

	Year	Unadjusted			Adjusted for Patient Characteristics			Adjusted for Patient and Physician Characteristics		
		Odds Ratio	95% CI	*p*-value	Odds Ratio	95% CI	*p*-value	Odds Ratio	95% CI	*p*-value
Income Q1 (lowest)										
Enhanced vs. traditional fee-for-service	2005	1.00	(0.98–1.02)	0.677	0.99	(0.97–1.01)	0.283	1.00	(0.98–1.02)	0.698
	2006	1.12	(1.10–1.14)	<.0001	1.11	(1.09–1.13)	<.0001	1.11	(1.10–1.13)	<.0001
	2007	1.24	(1.21–1.27)	<.0001	1.23	(1.20–1.26)	<.0001	1.24	(1.21–1.27)	<.0001
	2008	1.15	(1.12–1.19)	<.0001	1.16	(1.13–1.20)	<.0001	1.18	(1.14–1.22)	<.0001
	2009	1.26	(1.22–1.31)	<.0001	1.30	(1.25–1.35)	<.0001	1.29	(1.24–1.34)	<.0001
	2010	1.05	(1.00–1.10)	0.048	1.07	(1.02–1.12)	0.009	1.10	(1.04–1.15)	0.000
	2011	1.01	(0.96–1.08)	0.655	1.01	(0.95–1.08)	0.747	1.05	(0.99–1.12)	0.115
	2012	1.12	(1.05–1.20)	0.001	1.20	(1.12–1.29)	<.0001	1.20	(1.12–1.29)	<.0001
Income Q5 (highest)										
Enhanced vs. traditional fee-for-service	2005	0.99	(0.98–1.01)	0.190	1.00	(0.99–1.02)	0.895	1.00	(0.98–1.01)	0.853
	2006	1.05	(1.04–1.07)	<.0001	1.07	(1.05–1.08)	<.0001	1.08	(1.06–1.09)	<.0001
	2007	1.24	(1.21–1.26)	<.0001	1.25	(1.22–1.28)	<.0001	1.26	(1.23–1.29)	<.0001
	2008	1.22	(1.18–1.26)	<.0001	1.25	(1.21–1.29)	<.0001	1.28	(1.24–1.32)	<.0001
	2009	1.26	(1.21–1.30)	<.0001	1.30	(1.25–1.35)	<.0001	1.28	(1.24–1.34)	<.0001
	2010	1.19	(1.13–1.25)	<.0001	1.22	(1.15–1.28)	<.0001	1.24	(1.17–1.31)	<.0001
	2011	1.21	(1.13–1.30)	<.0001	1.23	(1.15–1.32)	<.0001	1.27	(1.18–1.36)	<.0001
	2012	1.17	(1.09–1.27)	<.0001	1.25	(1.16–1.36)	<.0001	1.26	(1.16–1.36)	<.0001
Ratios of Odds Ratios										
Difference in enhanced vs. traditional fee-for-service between Q1 vs. Q5	2005	1.01	(0.98–1.03)	0.619	0.99	(0.97–1.01)	0.344	1.00	(0.97–1.02)	0.847
	2006	1.06	(1.04–1.09)	<.0001	1.04	(1.02–1.06)	0.000	1.04	(1.01–1.06)	0.001
	2007	1.00	(0.97–1.03)	0.984	0.99	(0.95–1.02)	0.389	0.98	(0.95–1.02)	0.329
	2008	0.94	(0.90–0.99)	0.011	0.93	(0.89–0.97)	0.002	0.92	(0.88–0.97)	0.001
	2009	1.01	(0.95–1.06)	0.860	1.00	(0.95–1.06)	0.892	1.00	(0.95–1.06)	0.958
	2010	0.88	(0.82–0.95)	0.001	0.88	(0.82–0.94)	0.001	0.89	(0.82–0.95)	0.001
	2011	0.84	(0.77–0.92)	0.000	0.82	(0.75–0.90)	<.0001	0.83	(0.75–0.91)	<.0001
	2012	0.95	(0.86–1.06)	0.374	0.96	(0.87–1.07)	0.471	0.96	(0.86–1.06)	0.405

Results for Q2-Q4 not shown. Ratios of odds ratios are also presented, with Q5 and traditional fee-for-service serving as referent groups

Tables 6, 7, 8 also show the ratios of odds ratios comparing differences between patients living in the lowest and highest income quintiles between enhanced FFS and traditional FFS models. From 2005 to 2011, ratios of ratios were significantly less than 1 for breast and cervical cancer screening, indicating that the transition to enhanced FFS was associated with a widening of screening gaps between people of lowest and highest income. In 2012, ratios of ratios were no longer significantly different than 1 for breast cancer screening (ratio of odds ratios 0.966 [95% confidence interval 0.824–1.133] adjusted for patient and physician characteristics). For CRC screening, in contrast, ratios of odds ratios varied over the years. However, the transition to enhanced FFS tended to either be non-significant or to be associated with a widening of the gap in most years.

In 2002, when all physicians were still in traditional FFS practices, adjusted odds ratios comparing the lowest income to the highest income patients were 0.720 [95% confidence interval 0.712–0.728] for cervical screening 0.651 [95% confidence interval 0.639–0.663] for breast screening, and 0.817 [95% confidence interval 0.806–0.827] for CRC screening. In 2013, these values were 0.691 [95% confidence interval 0.682–0.701] for cervical cancer screening, 0.707 [95% confidence interval 0.692–0.723] for breast screening and dropped to 0.638 [95% confidence interval 0.629–0.648] for CRC screening.

## Discussion

We conducted a population-based longitudinal analysis from 2002 to 2013 among a group of family physicians that all eventually transitioned to enhanced FFS from traditional FFS in Ontario. We found that this transition of primary care model type was generally associated with increased screening uptake for both immigrants and long-terms residents, and for people in both the lowest and highest income quintiles. However, the transition appeared to be less beneficial for more disadvantaged groups, as it was associated with a widening of screening inequities for foreign-born and low-income patients, with the exception of the most recent years. Although the early adopters of the enhanced model had better overall screening rates to begin with, and continued this performance after transition, screening inequities were worse for patients of these physicians after adjusting for certain patient and physician characteristics. In contrast, the latest adopters tended to have lower overall screening rates but smaller screening inequities. These physicians saw an improvement in their overall screening with the shift to enhanced FFS. Of note, overall screening for CRC rose dramatically over time but this appears to be due to secular trends and not to the enhanced FFS transition [43].

It is likely that physicians with certain characteristics, or those treating patients of certain characteristics, were more likely to find the new models appealing and therefore to transition in earlier years. New models provided financial rewards for having high overall cancer screening rates, and did not incorporate equity measures into these rewards. It is not possible to determine the role that preventive care bonuses played for the physicians in this study. However, a previous Ontario study demonstrated that family physicians self-selected into the different model types, and suggested that physicians who had more patient visits, had a greater proportion of immigrants in their roster, and had patients with a higher level of need were more likely to select traditional FFS than enhanced FFS [44]. Of note, all the physicians in our study eventually transitioned to enhanced FFS and the screening patterns of those physicians who never transitioned, or who transitioned to other model types, are not known.

Our findings are comparable to other literature that has explored the impacts of primary care reform in Ontario on screening and preventive care. Kiran et al. found that in the year after financial incentives were introduced to primary care, there were no significant changes in breast and cervical screening and only a small increase in CRC screening; income-related inequalities persisted for all three cancer screening types [32]. Patients enrolled to one of the new primary care models were more likely to receive screening than other patients, even before the introduction of financial incentives [32]. Dahrouge et al. found that no particular model type was clearly associated with superior preventive care [45]. Physician characteristics, such as gender and patient load, were more predictive of performance [45]. In one cross-sectional analysis conducted in 2011, there were minimal differences in cancer screening between primary care model types, including enhanced FFS and capitation models [46]. Although primary care reform has not had a clear effect on screening and equity, the literature does suggest that immigrant and low-income patients who are in some type of PEM have higher screening rates than those who are not [15, 16, 21, 47]. However, a recent Ontario study showed that patients who are foreign-born and living in low-income neighbourhoods were more likely to still be in traditional FFS practices [48].

This study has several limitations. First, current administrative data do not include Pap tests interpreted in hospital laboratories and thus may underestimate cervical cancer screening. However, we expect that relatively few screening Pap tests are interpreted in the hospital setting and have no reason to believe that this would differ by primary care model type. Second, the IRCC database is not 100% sensitive, and does not include immigrants who moved to other provinces in Canada before moving to Ontario or who could not be probabilistically linked. Therefore, some immigrants would have been mistakenly included with long-term residents. Third, all patients prior to transition were attributed using virtual enrolment, whereas after transition, patients were attributed to physicians using a combination of both virtual and formal enrolment. Fourth, due to the complexity of the analytical models and the sheer size of the analytical dataset, it was not possible to account for heterogeneity among practices in their trends over time by including time as a random effect. Finally, our results cannot be considered generalizable to physicians or patients who transitioned from traditional FFS to other model types.

Future research should explore how more recent large-scale transitions (e.g. from enhanced FFS to primarily capitation-based models) have affected cancer screening and other measures of quality of care for marginalized groups. Importantly, some forms of capitation-based models emphasize team-based care, with non-physician health professionals such as nurse practitioners being integral parts of the primary care team, and it is feasible that the transition to these types of models may be associated with a decrease in screening gaps, as has been suggested for diabetes monitoring [30].

## Conclusion

In this study, we have found that the transition to enhanced FFS was not associated with a reduction in inequities. In fact, inequities generally tended to increase. We also found that early adopters of this new model had higher overall screening, but larger screening gaps for their foreign-born and low-income patients, than later adopters. It remains to be seen how other primary care transitions in Ontario have affected cancer screening gaps and other health inequities for immigrant and low-income patients.

## Abbreviations

CAPE: Client agency program enrolment
CRC: Colorectal cancer
FFS: Fee-for-service
ICES: Institute for clinical evaluative sciences
IRCC: Immigration, refugees and citizenship canada
OHIP: Ontario health insurance plan
PEM: Patient enrolment model
RDPB: Registered persons database
